# En face swept-source optical coherence tomography (SS-OCT) and SS-OCT angiography findings of retinal astrocytic hamartomas in patients with tuberous sclerosis complex

**DOI:** 10.3389/fmed.2025.1575006

**Published:** 2025-05-23

**Authors:** Chen-Xi Zhang, Kai-Feng Xu, Qin Long, Xiao Zhang, Zhi-Kun Yang, Rong-Ping Dai, Zhi-Qiao Zhang

**Affiliations:** ^1^Department of Ophthalmology, Peking Union Medical College Hospital, Chinese Academy of Medical Sciences and Peking Union Medical College, Beijing, China; ^2^Key Laboratory of Ocular Fundus Diseases, Chinese Academy of Medical Sciences, Beijing, China; ^3^Beijing Key Laboratory of Fundus Diseases Intelligent Diagnosis & Drug/Device Development and Translation, Beijing, China; ^4^Department of Pulmonary and Critical Care Medicine, State Key Laboratory of Complex Severe and Rare Diseases, Peking Union Medical College Hospital, Chinese Academy of Medical Sciences and Peking Union Medical College, Beijing, China

**Keywords:** en face, swept source, optical coherence tomography angiography, retinal astrocytic hamartoma, tuberous sclerosis complex

## Abstract

**Introduction:**

Tuberous sclerosis complex (TSC) is an autosomal dominant disorder characterized by multisystem hamartomas, including retinal astrocytic hamartomas (RAHs), which are a key diagnostic criterion. This study evaluates the en face swept-source optical coherence tomography (SS-OCT) and SS-OCT angiography (SS-OCTA) features of TSC-associated RAHs.

**Methods:**

A retrospective analysis of 10 patients with TSC-associated RAHs was conducted using en face SS-OCT, SS-OCTA, and fundus photography. Structural and vascular features of the lesions were assessed based on these imaging modalities.

**Results:**

Of the 10 TSC patients, 21 RAH lesions (18 type 1, 1 type 2, 2 type 3) were completely scanned. En face SS-OCT revealed vitreous changes in 17 of the 21 RAH lesions, with clear visualization of vitreoretinal traction in 6 lesions. Type 1 RAHs appeared as isoreflective or mildly hyporeflective masses with disarrangement of retinal nerve fibers. Calcified components in type 2 or type 3 RAHs appeared differently on the en face choroid slab, with type 2 RAHs featuring closely arranged isoreflective vesicles, while type 3 RAHs appeared as sharply defined dark areas. On SS-OCTA, a dense vascular network with disorganization of the radial papillary capillaries was observed in almost all of the type 1 RAHs, with half of them exhibiting congestive intrinsic microvasculature. Feeder vessels were identified in only two type 1 lesions. Non-flow, moth-eaten cavities were characteristic of the calcified components of type 2 or type 3 RAHs. The tumor vascular density was positively correlated with tumor maximal thickness in type 1 RAHs (*p* = 0.037).

**Conclusion:**

En face SS-OCT provided a good display of RAH-related vitreoretinal traction and tumor calcification, while SS-OCTA, by clearly visualizing intratumoral vascularity, may assist in detecting signs of progressive tumor growth in TSC-associated RAHs.

## 1 Introduction

Tuberous sclerosis complex (TSC) is an autosomal dominant disorder caused by mutations in either the TSC1 or TSC2 genes. This genetic condition is characterized by multisystem hamartomas, which involve the brain, heart, lungs, kidneys, skin, and eyes (1). Retinal astrocytic hamartomas (RAHs), which are benign tumors primarily composed of astrocytes, stand out as one of the major diagnostic criteria for TSC. RAHs are notably the most common ophthalmic finding in TSC, with an occurrence rate of 75.8% among Chinese patients with TSC, as reported in our previous study (2–4).

RAHs are clinically classified into three groups based on ophthalmoscopic manifestations: type 1 RAHs are non-calcified, gray-white translucent masses, occasionally containing intralesional cavities; type 2 RAHs feature a mulberry-like appearance, being fully composed of calcified nodules; and type 3 RAHs are partially calcified and considered transitional lesions, combining features of the two previous types (5). The spectral domain optical coherence tomography (OCT) features of the three types of TSC-associated RAHs have been described in previous studies, ranging from the thickening of the retinal nerve fiber layer (RNFL) with retinal disorganization to classic moth-eaten appearance with posterior dense optical shadowing (3, 6, 7).

Although the majority of RAHs remain stable over the years, progressive tumor growth resulting in macular edema and neovascular glaucoma has been reported, suggesting a vasogenic association in RAHs (8). Optical coherence tomography angiography (OCTA), as an emerging non-invasive imaging technique, enables the examination of vascular features of retinal lesions, while en face OCT provides a frontal plane perspective, allowing for layer-specific retinal visualization. Although several case reports have described the en face OCT and OCTA characteristics of RAHs, the majority focused on type 1 RAHs that were not associated with TSC (9–14). Notably, certain characteristics, such as the central feeder vessel of type 1 RAHs identified by Yung et al. using spectral-domain OCTA, were not observed in other cases (11).

Moreover, the swept-source OCT (SS-OCT) system, distinguished by its long wavelength and high scanning speed, surpasses the spectral-domain OCT (SD-OCT) system by offering deeper imaging penetration and minimizing motion artifacts. This results in enhanced visualization of intratumoral configurations, such as RAHs, which is essential for their accurate diagnosis and monitoring. In this study, we evaluated the en face SS-OCT and SS-OCT angiography (SS-OCTA) findings of TSC-associated RAHs in a retrospective case series, aiming to highlight the diagnostic and monitoring advantages of SS-OCT in these complex cases.

## 2 Materials and methods

### 2.1 Participants

We reviewed the en face SS-OCT and SS-OCTA imaging of patients with the diagnosis of TSC-associated RAHs from 1 December 2019 to 31 December 2023, at the Department of Ophthalmology, Peking Union Medical College Hospital, Peking, China. The diagnosis of TSC was established based on the 2012 TSC diagnostic criteria or positive results of genetic testing, and RAHs were detected and classified into three types ophthalmologically. Demographic information, including gender and age at the ophthalmic examination, along with ocular past history, including treatment, was collected from each patient. Ethical approval to undertake this study was obtained from the ethical committee of Peking Union Medical College Hospital (Approved number: I-22PJ993). The research adhered to the tenets of the Declaration of Helsinki.

### 2.2 Data collection

En face SS-OCT and SS-OCTA imaging of the tumor lesion were acquired using an SS-OCT system (VG200D, SVision Imaging, Ltd., China). This swept-source system has a central wavelength of 1,050 nm and a scanning rate of 200,000 A-scans per second. Depending on the location and size of RAH lesions, macular raster scans or optic nerve head raster scans of 3 × 3 mm, 6 × 6 mm, 9 × 9 mm were used. The custom segmentation capability of the device allows for the visualization of specific anatomical layers. For en face SS-OCT, the vitreous, RNFL, and choroid layers were transected for analysis. For SS-OCTA, the radial peripapillary capillary (RPC) slab extended from 5 μm above the internal limiting membrane to the interface between RNFL and ganglion cell layer. The deep capillary plexus slab was generated from the middle of the inner nuclear layer (INL) to 25 μm beneath the INL/outer plexiform layer (OPL). The outer retina was segmented from 25 μm beneath the INL/OPL to 10 μm above the retinal pigment epithelial layer, and the choriocapillaris was segmented from 10 μm above Bruch’s membrane to 25 μm below it. Segmentation errors were checked and manually corrected before interpreting en face images. In addition, fundus findings, including tumor location by quadrant, were recorded by fundus photography (TRC NW6S, Topcon Corp, Japan) or ultra-wide-field scanning laser ophthalmoscopy (Daytona, Optos PLC, Dunfermline, UK). Tumor maximal thickness, defined as the distance from the highest peak of the anterior surface of a tumor to the retinal pigment epithelium, was measured on SS-OCT B-scan, and the lesion area, well delineated by the thickening of RNFL in RAHs, was measured on SS-OCT retinal thickness maps. Scans with a signal strength below 7, as indicated by the device’s output and those with significant image artifacts, were excluded from the analysis.

### 2.3 Statistical analysis

The RPC slabs of SS-OCTA images were overlaid on the retinal thickness maps to assess the vascularization within RAH lesions. Vascular density within the lesion was computed after binarization of the RPC using Image J (ImageJ 2.3.0). The correlation between tumor vascular density and tumor maximal thickness or tumor area was studied using Spearman’s correlation analysis. A *p*-value of < 0.05 was considered statistically significant.

## 3 Results

Ten TSC patients (four men and six women) with RAHs were included in the study. The mean age at referral was 30.6 ± 9.0 years (range, 17–44; median, 30). A total of 21 RAHs were completely scanned using SS-OCT and SS-OCTA in 13 eyes of 10 patients, of which 18 lesions were type 1 RAHs, only 1 lesion was type 2 RAHs, and 2 lesions were type 3 RAHs. The majority of RAHs were located in the inferotemporal quadrant (9/21), followed by the superotemporal (7/21) and inferonasal quadrants (4/21). The type 2 RAHs were juxtapapillary. The mean tumor area was 4.34 ± 4.03 mm^2^ (range, 0.76–16.51; median, 3.33), and the mean tumor maximal thickness was 566.1 ± 170.1 μm (range, 247–947; median, 512). Of the 13 affected eyes, 2 had a history of vitreous hemorrhage, which resolved or cleared after laser monotherapy or vitrectomy combined with anti-vascular endothelial growth factor (VEGF) therapy, making them eligible for SS-OCT and SS-OCTA evaluation. The en face SS-OCT and SS-OCTA findings for TSC-associated RAHs are summarized in Table 1.

**Table 1:** Summary of en face swept-source optical coherence tomography (SS-OCT) and SS-OCT angiography(SS-OCTA) findings of retinal astrocytic hamartomas (RAHs) in tuberous sclerosis complex(TSC) patients.

Imaging	Type 1 RAHs	Type 2 RAHs^*^	Type 3 RAHs^*^
En face SS-OCT			
Vitreous	- homogenous mild hyperreflectivity (abnormal vitreoretinal adhesion) - focal irregular hyperreflectivity (vitreous thickening or condensation) - hyperreflective spots (interdigitations with vitreous) - patterns of vitreoretinal traction		
Retinal nerve fiber layer	- isoreflective or mild hyporeflective mass with disarrangement of retinal nerve fiber layer -multiple intratumoral hyporeflective cavities	- heterogenous reflective appearance with less well-delineated multinodular appearance (calcified components)	
Choroid	- normal or mild hyporeflective appearance - intratumoral microstructures * shadows of intratumoral vessels * large hyperreflective intratumoral cavity due to increased light transmission	- well-illustrated mulberry-like appearance - multinodules shown as closely arranged isoreflective vesicles with hyporeflective rings	- different from type 2, calcified component shown as sharply defined dark area
SS-OCTA			
Radial peripapillary capillary	- dense vascular network with disorganized radial peripapillary capillaries - intratumoral microstructures * curled and intertwined congestive microvasculature (9/18 lesions) * feeder vessel (2/18 lesions) * low flow signal in intratumoral cavities (2/18 lesions)	- sparse vascular network - flow localized to walls surrounding cavities	- lower vascular density in the central calcified region compared to the surrounding areas
Deep capillary plexus	- irregular hyporeflective changes with varied projection artifacts	- hyporeflectivity	-dark appearance
Outer retina	- normal or mild hyporeflectivity	- hyporeflectivity	-dark appearance
Choriocapillaris	- normal or mild hyporeflectivity	- mixed reflectivity	-dark appearance

^*^The findings for type 2 and type 3 RAHs are based on a limited number of cases.

### 3.1 En face SS-OCT

Vitreous changes were present in 17 RAH lesions. At the vitreous segmentation of en face SS-OCT, homogenous mild hyperreflectivity, referring to abnormal vitreoretinal adhesion in B-scan OCT, was found in 11 RAHs. Focal irregular hyperreflectivity corresponding to the area of vitreous thickening or condensation was observed in five RAHs. Three RAH lesions had apparent interdigitations with vitreous, which were observed as hyperreflective spots on en face images. Vitreoretinal traction was found in six RAHs, a pattern well-demonstrated by en face SS-OCT imaging.

**Figure 1:**
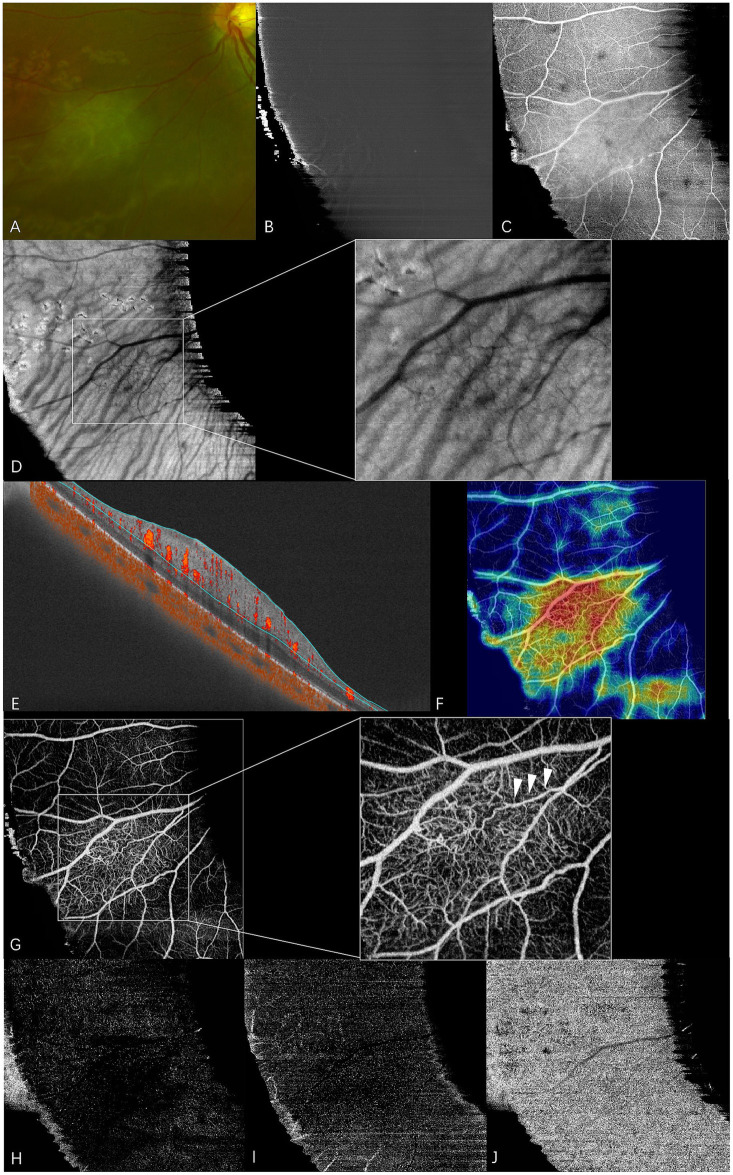
En face swept-source optical coherence tomography (SS-OCT) and SS-OCT angiography (SS-OCTA) of type 1 RAHs in TSC patient 9. The patient had an ocular history of vitreous hemorrhage in this eye and received vitrectomy combined with anti-VEGF therapy 2 years before the examination. Focal retinal photocoagulation was performed during the surgery. **(A)** An ultra-wide-field scanning laser ophthalmoscope image (part) shows gray-white semitransparent type 1 RAH lesions and surrounding depigmented laser spots. **(B–D)** en face SS-OCT. The lesion appears normal at the vitreous slab **(B)**, and isoreflective disorganized retinal nerve fibers are shown at the level of the retinal nerve fiber layer (RNFL) **(C)**. Shadows of intratumoral vessels are observed in the choroid slab with local magnification **(D)**. **(E–J)**. SS-OCTA (9 × 9 mm scan). Angio B-scan with flow overlay and radial peripapillary capillary (RPC) segmentation lines maps the flow mainly to the thickening RNFL **(E)**, and the heatmap of vascular density demonstrates the increased vascular density within the lesion **(F)**. RPC slab of SS-OCTA clearly shows a dense vascular network with congestive intrinsic microvasculature, and the central feeder vessel is identified by arrows at the local magnification **(G)**. The lesion appears mild hyporeflective at the segmentation of deep capillary plexus **(H)** and outer retina **(I)**, while almost normal at the choriocapillaris segmentation **(J)**.

**Figure 2:**
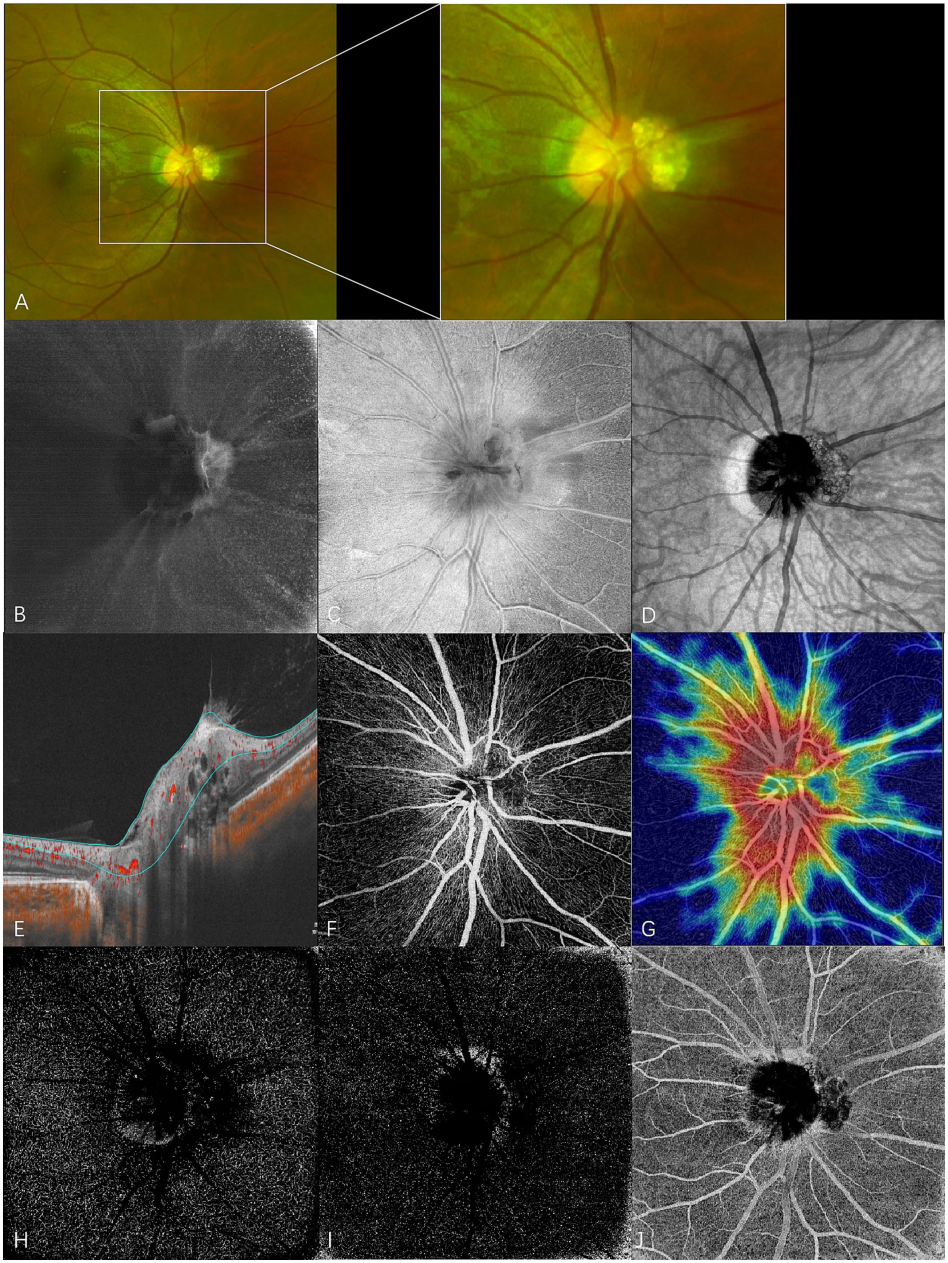
En face swept-source optical coherence tomography (SS-OCT) and SS-OCT angiography (SS-OCTA) of type 2 RAHs in TSC patient 4. **(A)** An ultra-wide-field scanning laser ophthalmoscope image (part) with local magnification shows juxtapapillary type 2 RAHs with a mulberry-like appearance. **(B–D)** en face SS-OCT. At the level of vitreous, irregular hyperreflectivity indicating the abnormal interaction between the lesion and vitreous is shown, and vitreoretinal traction on the lesion leads to the formation of radiating folds of the posterior vitreous cortex around the optic disk **(B)**. The lesion is poorly defined, and the multi-calcified nodules appear isoreflective or hyporeflective at the level of the retinal nerve fiber layer (RNFL) **(C)**. The mulberry-like appearance of type 2 RAHs is well presented at the segmentation of the choroid **(D)**, shown as closely arranged isoreflective vesicles demarcated by hyporeflective rings. **(E–J)** SS-OCTA (ONH scan). Angio B-scan with flow overlay and radial peripapillary capillary (RPC) segmentation lines maps the flow to the walls surrounding moth-eaten cavities **(E)**. RPC slab of SS-OCTA shows that the lesion has a sparse vascular network **(F)**, and the vascular density is significantly low compared to the surroundings in the heatmap for RPC vascular density **(G)**. Hyporeflective changes are shown at the segmentation of the deep capillary plexus **(H)** and outer retina **(I)**, while mixed reflectivity is detected at the choriocapillaris segmentation **(J)**.

**Figure 3:**
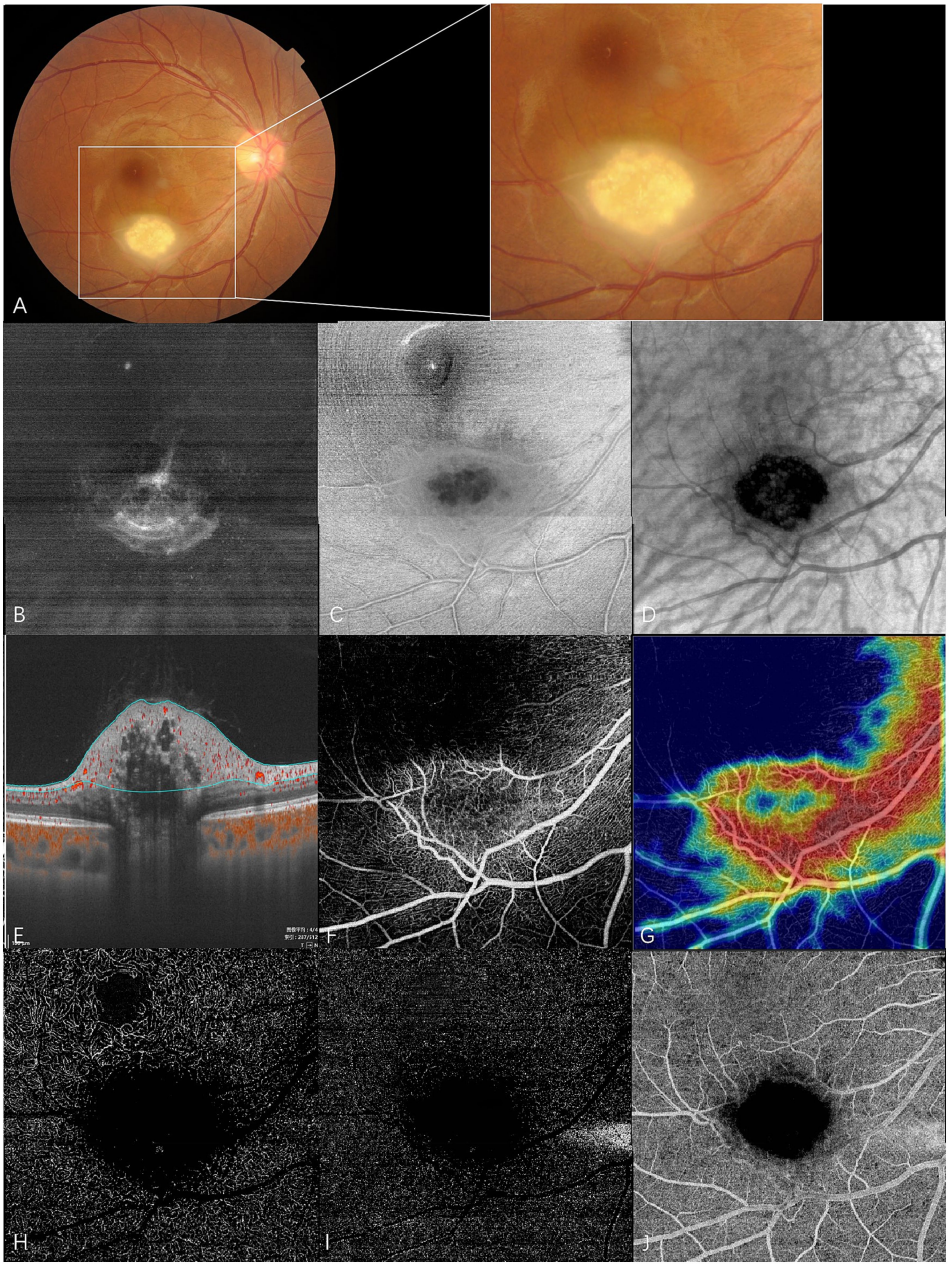
En face swept-source optical coherence tomography (SS-OCT) and SS-OCT angiography (SS-OCTA) of type 3 RAHs in TSC patient 2. **(A)** Color fundus photography with local magnification shows type 3 RAHs, an oval, gray-white semitransparent lesion with central calcifications, located at the inferotemporal arcade. **(B–D)** en face SS-OCT. At the level of vitreous, irregular hyperreflectivity representing vitreous thickening is shown, and radiating vitreoretinal traction on the lower part of the lesion is demonstrated **(B)**. The lesion appears ill-defined with peripheral isoreflectivity, and central calcifications are unevenly hyporeflective at the level of the retinal nerve fiber layer (RNFL) **(C)**. At the level of the choroid, central calcifications appear dark and well-delineated, while several isoreflective vesicles are detected within the dark area **(D)**. **(E–J)** SS-OCTA (6 × 6 mm). Angio B-scan with flow overlay and radial peripapillary capillary (RPC) segmentation lines maps the flow mainly to the peripheral non-calcified component. No flow is detected within central empty optical spaces **(E)**. The RPC slab of SS-OCTA shows the lesion that has an annular dense vascular network **(F)**, and the vascular density is much lower in the central feeder as illustrated by the heatmap of vascular density at the RPC slab **(G)**. The lesion appears dark at the segmentation of the deep capillary plexus **(H)**, outer retina **(I)**, and choriocapillaris **(J)**, possibly due to the shadowing effect.

RAHs developed from the RNFL. On the RNFL slab of en face SS-OCT, type 1 RAHs presented as isoreflective or mildly hyporeflective masses with disarrangement of the retinal nerve fibers (Figure 1C). Multiple intratumoral cavities were detected within one type 1 RAH lesion and were observed as hyporeflective on en face images. Calcified components of type 2 or type 3 RAHs appeared heterogenously reflective, and the multinodular appearance was less well-delineated on the RNFL slab, likely due to the varied depth localization of calcifications and optically empty nodules (Figures 2C, 3C). It is important to mention that, in previous OCT B-scan studies, both intratumoral calcification and cavitation, presenting with moth-eaten appearances on OCT, were identified as optically empty spaces (OESs), without further differentiation between these two conditions (6, 15).

At the en face choroid slab, half of the type 1 RAHs (9/18) appeared normal. Mild hyporeflectivity caused by overlying thickened RNFL was detected in seven type 1 lesions. Shadows of intratumoral vessels were identified in two type 1 RAHs, and scattered hyporeflective spots representing microcalcifications were observed in one type 1 RAH (Figure 1D). The largest intratumoral cavity within type 1 RAHs appeared hyperreflective, possibly due to increased light transmission into the choroid, where the solid tissue had been replaced by empty spaces. The mulberry-like appearance of type 2 RAHs was well illustrated using a choroid slab. Multinodules were observed as closely arranged isoreflective vesicles, which were demarcated by hyporeflective rings (Figure 2D). The isoreflective vesicles co-localized with optically empty cavities, while the hyporeflective rings resulted from posterior dense shadowing of the walls surrounding the cavities, as observed in the corresponding B-scans. The calcified component of type 3 RAHs presented differently from that of type 2 RAHs at the level of the choroid. The sharply defined dark area refers to the calcified component in type 3 RAHs. Several isoreflective vesicles were identified within the dark area in one type 3 lesion (Figure 3D).

### 3.2 SS-OCTA

The RPC, co-located with the RAH lesions within the RNFL, was visualized using en face OCTA segmentation. Almost all type 1 RAHs exhibited a dense vascular network with disorganized RPCs, except for one peripapillary lesion overlaying a superotemporal retinal artery branch. Congestive intrinsic microvasculature was well recognized in half of the type 1 RAHs (9/18), presented as curled and intertwined vessels. A feeder vessel was clearly identified only in two type 1 RAHs, and intratumoral cavities within type 1 RAH lesions showed a low flow signal. The average vascular density of type 1 RAHs at the level of RPC was 36.2 ± 9.1% (range, 22.2–55.8; median, 37.3). A positive correlation was observed between the vascular density and maximal thickness of type 1 RAH lesions (r = 0.510, *p* = 0.037, Spearman’s correlation analysis). No significant correlation was found between the vascular density and the tumor area (*p* = 0.086, Spearman’s correlation analysis). At the deep capillary plexus segmentation, type 1 RAHs exhibited irregular hyporeflective changes with varied projection artifacts, while outer retinal and choriocapillaris segmentations appeared normal or mildly hyporeflective (Figures 1E–J).

Type 2 RAHs were shown to have a sparse vascular network at the RPC segmentation. Flow overlay on the corresponding B-scans localized flow to the walls surrounding the moth-eaten cavities. The vascular density of this lesion at the level of RPC was 40.2%. Hyporeflective changes were detected at the deep capillary plexus and outer retina segmentations, while the choriocapillaris segmentation showed mixed reflectivity (Figures 2E–J).

Type 3 RAHs exhibited significantly lower vascular density in the central calcified region compared to the surrounding areas at RPC slab segmentation. The vascular density of the whole lesion at RPC was 53.0% and 43.8%, respectively. The tumors appeared dark at the deep capillary plexus, outer retina, and choriocapillaris segmentations (Figures 3E–J).

## 4 Discussion

We described the en face SS-OCT and SS-OCTA in 21 TSC-associated RAHs, involving three types of RAHs. Distinct differences in characteristics were shown among the three types. En face SS-OCT offers frontal, layer-specific visualization that enhances the assessment of vitreoretinal traction, intratumoral cavities, and calcifications in RAHs, which may not be readily appreciated on conventional cross-sectional B-scans. The ability to detect the pattern of vitreoretinal traction is particularly significant, as previous studies have suggested an association between tractional changes and vitreous seeding in RAHs (16, 17), similar to the dissemination of retinoblastoma. En face illustration of vitreous traction, therefore, may help in the early detection of vitreous seeding.

Despréaux et al. (13) described the value of en face OCT findings in RAHs and expressed a negative opinion about its value in RAHs, believing that it provided limited useful information, except for better delineation of lesions. However, in our study, disarrangement of retinal nerve fibers was observed in the RNFL slab of en face SS-OCT, and the choroidal slab revealed more details on intratumoral changes, including shadowing/masking or transillumination effects from overlying structures. OESs, first described by Shields et al. on time-domain OCT with characteristic moth-eaten appearances, represent intralesional calcification or cavitation (6, 15). To date, the significance of OESs has not been completely clarified, and histopathological evidence on OESs in RAHs is still lacking (15). In our study, OESs showed different en face SS-OCT patterns on choroid slabs: Hyperreflectivity was detected for OESs within cavitary RAHs, while type 2 RAH OESs were observed as closely arranged isoreflective vesicles demarcated by hyporeflective rings. Although the calcified components (OESs) of type 3 RAHs looked similar to type 2 RAHs ophthalmoscopically or on B-can OCT, type 3 RAHs displayed more pronounced shadowing/masking effects and appeared predominately dark at the choroid segmentation of en face SS-OCT, which may suggest histopathological differences among intralesional calcified OESs. Moreover, further histopathological studies should be conducted to verify this. Considering that calcification progression in TSC-associated RAHs has been documented over time (4), en face SS-OCT may help in monitoring the progression from an optical perspective, besides differentiating cavitary OESs from the calcified ones.

SS-OCTA revealed the intrinsic vascularity of RAHs. Yung et al. (11) were the first to reported the OCTA features of type 1 RAHs, identifying a central feeder vessel within the tumor, whereas in a study by Despréaux et al. (13), a dense vascular network instead of a feeder vessel was described. In our study, feeder vessels were only detected in two type 1 RAHs, while a dense vascular network with RPC disorganization was demonstrated in almost all type 1 RAHs.

This research is the first of its kind to report on SS-OCTA findings in TSC-associated types 2 and 3 RAHs. We noted non-flow moth-eaten cavities in the RPC layer and sparse vascular flow along cavity walls on SS-OCTA B-scans. Hyporeflective changes were found at the deep capillary plexus, outer retina, and choriocapillaris segmentations due to shadowing/masking effects from calcification in type 2 RAHs. Type 3 RAHs combined the SS-OCTA features of type 1 and type 2 RAHs, with central hyporeflectivity representing the calcified area and peripheral hyperreflectivity representing the surrounding vascular network at RPC segmentation. However, the limited sample size of type 2 and type 3 RAHs highlighting the need for larger studies to confirm and expand upon these findings.

Using Image J software, we calculated vascular density within RAH lesions from RPC flow images, finding a medium vascular density ranging from 22.2 to 55.8%. A positive correlation was observed between the vascular density on SS-OCTA RPC segmentation and the maximal thickness on SS-OCT B scans in type 1 RAH lesions. Tumor maximal thickness, widely used as the main measurement to evaluate the size of RAH lesions, was reported to be significantly reduced in the treatment of mammalian targets with rapamycin (mTOR) inhibitors for TSC (18–20). To date, no studies have followed up TSC-associated RAHs using OCTA in mTOR inhibitor treatment. Amoroso et al. (21) previously reported stable vascular density on superficial capillary plexus (SCP) segmentation in one case of untreated isolated type 1 RAHs that was followed up using OCTA for 2 years. We propose that OCTA may serve as a more sensitive modality to assess the efficacy of mTOR inhibitors for TSC-associated RAHs, considering that blood flow is essential for tumor growth and that changes in RPC or SCP vascular density may precede the changes in maximal thickness of RAHs. For TSC patients undergoing mTOR inhibitor therapy, SS-OCTA imaging of RAHs may further serve as a surrogate marker for systemic disease activity in TSC, where structural changes in TSC-related brain or renal lesions are slow to manifest, and frequent imaging (e.g., brain MRI) is often impractical.

To our knowledge, this is the first description of en face SS-OCT and SS-OCTA characteristics of all three types of TSC-associated RAHs in one study. The great tissue penetration of the SS-OCT system facilitated the visualization of intratumoral configuration (9). RPC, instead of frequently used SCP, was specially segmented for SS-OCTA interpretation of RAH lesions, which was co-localized with RAHs in RNFL. OESs appeared differently in the choroid slab of en face SS-OCT, and quantitative analysis found a correlation between vascular density and increased tumor maximal thickness in type 1 RAHs, highlighting the potential utility of these imaging modalities in the follow-up of TSC-associated RAHs.

The limitations of this study include its small sample size and observational nature. Only one type 2 RAH and two type 3 RAH lesions were involved in this study, which limits the generalizability of our findings. However, as noted in our previous studies (3), type 2 and type 3 RAHs are relatively rare among Chinese TSC patients. Despite the limited number of cases, the inclusion of these cases offers valuable preliminary insights into the structural and vascular characteristics of rare RAH subtypes. Additionally, the absence of longitudinal data has limited the ability to reveal temporal changes related to en face SS-OCT and SS-OCTA characteristics of TSC-associated RAHs. There is a critical need for future studies, particularly those with larger cohorts, to explore the role of en face SS-OCT or SS-OCTA in monitoring the longitudinal effects of mTOR inhibitors on TSC-associated RAHs. These studies should also deepen our understanding of RAH progression and its response to treatment over time. These imaging modalities may hold promise not only for ocular assessment but also as surrogate markers for systemic treatment monitoring in TSC. Finally, while this study focused exclusively on TSC-associated RAHs, it is important to note that RAHs can also occur in other contexts, such as neurofibromatosis (NF) or as isolated findings. Therefore, our findings may not be generalizable to RAHs arising from other systemic conditions.

In summary, TSC-associated RAHs were well characterized using en face SS-OCT and SS-OCTA. En face SS-OCT had an advantage in the illustration of RAH-related vitreoretinal traction and reflection on the calcification of OESs, facilitating early detection of vitreous seeding and monitoring the progression of calcification in RAH lesions. Characteristic features of RAHs on SS-OCTA include RPC disorganization, a dense vascular network with or without congestive intrinsic microvasculature, and non-flow moth-eaten cavities. The feeder vessel was an uncommon finding in this study. SS-OCTA imaging may assist in detecting signs of RAH-associated vitreous hemorrhage and neovascularization. Additionally, tumor vascular density, which is correlated with tumor thickness, may provide a quantitative approach to follow up and evaluate the effects of mTOR inhibitors on TSC-associated RAHs.

## Data Availability

The original contributions presented in the study are included in the article/supplementary material, further inquiries can be directed to the corresponding author.
